# The effects of different ischemic conditioning on strength training recovery

**DOI:** 10.5114/biolsport.2025.144410

**Published:** 2024-11-05

**Authors:** Fan Zihan, Fu Yanqing, Wu Ying

**Affiliations:** 1 Beijing Sport University, No. 48 Xinxi Road, Haidian District, Beijing, P. R. China; 2 Laboratory of Sports Stress and Adaptation of General Administration of Sport, No. 48 Xinxi Road, Haidian District, Beijing, P. R. China

**Keywords:** Blood flow restriction, Ischemic preconditioning, Post-exercise ischemic, conditioning, Fatigue recovery, Strength training

## Abstract

The aim was to explore the impact of ischemic conditioning (IC) before or after strength training (ST) on recovery and to compare IC with traditional recovery methods (static stretching and foam rolling). Thirtyseven healthy males were divided into four groups: CON (no intervention), TRA (stretching and foam rolling after ST), IPC (IC before ST), and PEIC (IC after ST). The ST protocol consisted of five sessions, spaced every two days. Muscle soreness, thigh circumference (TC), countermovement jumps (CMJ), knee isokinetic muscle strength (peak torque [PT], relative peak torque [RPT]), and lactate dehydrogenase (LDH), creatine kinase (CK), malondialdehyde (MDA), superoxide dismutase (SOD), total antioxidant capacity (T-AOC), C-reactive protein (CRP) and interleukin-6 (IL-6) were measured at baseline, 24 h after the first intervention (1^st^–24 h), and 24 h and 48 h after the fifth intervention (5^th^–24 h, 5^th^–48 h). No significant differences were found in CMJ in PEIC at all timepoints (P > 0.05), while IPC had lower CMJ at 1^st^–24 h than baseline (P < 0.05). Right quadriceps RPT and PT in TRA were unchanged at all timepoints (P > 0.05), whereas IPC and PEIC had lower values at 1^st^–24 h than baseline (P < 0.05). No significant differences were found in LDH and IL-6 in IPC and PEIC at all timepoints (P > 0.05), but TRA showed significant differences in LDH at 1^st^–24 h and in IL-6 at 1^st^–24 h and 5^th^–24 h than baseline (P < 0.05). Results indicated acute PEIC better maintained CMJ than IPC. Acute TRA promoted faster recovery of lower extremity strength than IC, while IC led to a faster recovery of muscle damage and inflammation than TRA.

## INTRODUCTION

Elite athletes frequently face the challenges of intensive training sessions or multi-day competitions with short recovery periods. These activities induce physiological [1] and psychological [2] changes such as muscular damage, oxidative stress, and inflammatory responses, which can lead to a temporary decrease in performance [1, 3, 4]. As a result, athletes and coaches are exploring a variety of post-exercise recovery strategies [5] to improve exercise performance.

Ischemic conditioning (IC) is a process involving the application of short, intermittent periods of vascular occlusion followed by reperfusion. Initially utilized in clinical medicine, IC has been shown to be effective in protecting tissues from contractile and metabolic damage caused by ischemic reperfusion injury (IRI), whether applied before or after an ischemic event [6]. As research has progressed, the scope of IC’s applications has expanded significantly. Recent studies indicate that IC holds substantial promise in the field of sports science. Specifically, emerging research has highlighted the potential of IC applied before (ischemic preconditioning [IPC]) or following (post-exercise ischemic conditioning [PEIC]) exercise in expediting the recovery process after exercise [7–14]. A study indicated that repeated IPC application over several days was more effective than a single application in accelerating the recovery of knee maximal voluntary isometric contraction (MVIC), and both reduced swelling when compared with the sham condition [7]. PEIC also exhibited promise. A study found that PEIC improved muscle force recovery, reduced muscle soreness, and lowered CK activity after exercise-induced muscle damage (EIMD) [12]. However, some studies have shown that IPC and PEIC are not effective in promoting post-exercise fatigue recovery. For instance, Northy et al. [15] found that PEIC did not enhance fatigue recovery following resistance training. Similarly, Garcia et al. [16]reported that IPC failed to facilitate fatigue recovery in rugby players. Therefore, further research is needed to validate the effectiveness of IPC and PEIC in promoting fatigue recovery. Besides, few studies have directly compared IPC with PEIC. Nevertheless, the contrast between the pro-recovery effects of IPC and PEIC can not only enhance comprehension of the mechanism of IC but also facilitates its application in diverse sporting contexts. Thus, the aim of this study was to investigate the effects of IPC and PEIC on exercise-induced fatigue recovery and to compare the efficacy of these two methods. Moreover, there was a lack of studies comparing the effects of IC with traditional recovery methods, such as static stretching and foam rolling. Hence, the secondary goal was to assess and compare the differences between IC and traditional recovery methods (static stretching combined with foam rolling). The hypothesis posited that IC applied after ST was expected to attenuate exercise-induced fatigue during recovery to a greater extent than IC applied before ST. Additionally, both IPC and PEIC were hypothesized to yield more favorable outcomes compared to traditional recovery methods. Furthermore, both acute and repeated IC could accelerate the recovery process, with the repeated applications exerting a more pronounced effect.

## MATERIALS AND METHODS

### Participants

Thirty-seven healthy males with strength training experience (at least 1 year) volunteered for the study. Participants were physically active, non-smokers, and free of musculoskeletal injury. They did not report the use of dietary supplements or any medications that would contraindicate intense physical training. No participants had previously used IPC or PEIC. Participants were instructed to refrain from any form of exercise for at least 48 hours prior to their testing visits to avoid any residual fatigue effects. Before conducting the study, the Sports Science Experiment Ethics Committee of Beijing Sport University granted permission (approval No.: 2023072H), and all participants provided written informed consent and were informed of the study’s nature. All procedures were in accordance with the ethical standards of the Declaration of Helsinki, as revised in 2013. Testing was performed using G*Power 3.1 software, following the recommendations of Beck (2013) to reduce the probability of type II error and to determine the minimum number of participants required for this investigation. The parameters adopted for the sample size calculation were as follows: an effect size (f) of 0.25, a significance level (α) of 0.05, and a desired statistical power (1-β) of 0.80. Considering the study design with four groups and four measurement time points, a correlation among repeated measures of 0.5, and assuming sphericity (ε = 1), the minimal required sample size calculated in the power analysis was 36 participants (9 per group). Participant’s basic characteristics are presented in Table 1. There were no significant differences in age, weight, height, BMI, weekly activity time, absolute and relative 1RM of squat among the groups (all *P* > 0.05).

**TABLE 1:** Physical characteristics of the participants.

Variable	CON (n = 9)	TRA (n = 9)	IPC (n = 9)	PEIC (n = 10)	*p* value
Age (y)	20.2 ± 1.0	21.4 ± 1.3	20.4 ± 1.2	21.3 ± 0.8	0.055
Height (cm)	181 ± 6	179 ± 4	178 ± 5	179 ± 8	0.734
Weight (kg)	77.9 ± 9.8	73.3 ± 7.3	76.1 ± 9.8	76.1 ± 9.5	0.769
BMI (kg/m^2^)	23.7 ± 2.7	22.8 ± 1.9	23.9 ± 2.8	23.6 ± 2.2	0.760
Activity time (h/week)	9.9 ± 0.6	10.4 ± 2.7	11.1 ± 2.3	11.9 ± 2.5	0.342
1RM (kg)	101 ± 13	107 ± 17	115 ± 24	114 ± 19	0.232
Relative muscle strength	1.31 ± 0.15	1.46 ± 0.26	1.52 ± 0.30	1.51 ± 0.27	0.253

Values are presented as mean± SD.

### Study design

**FIG. 1:**
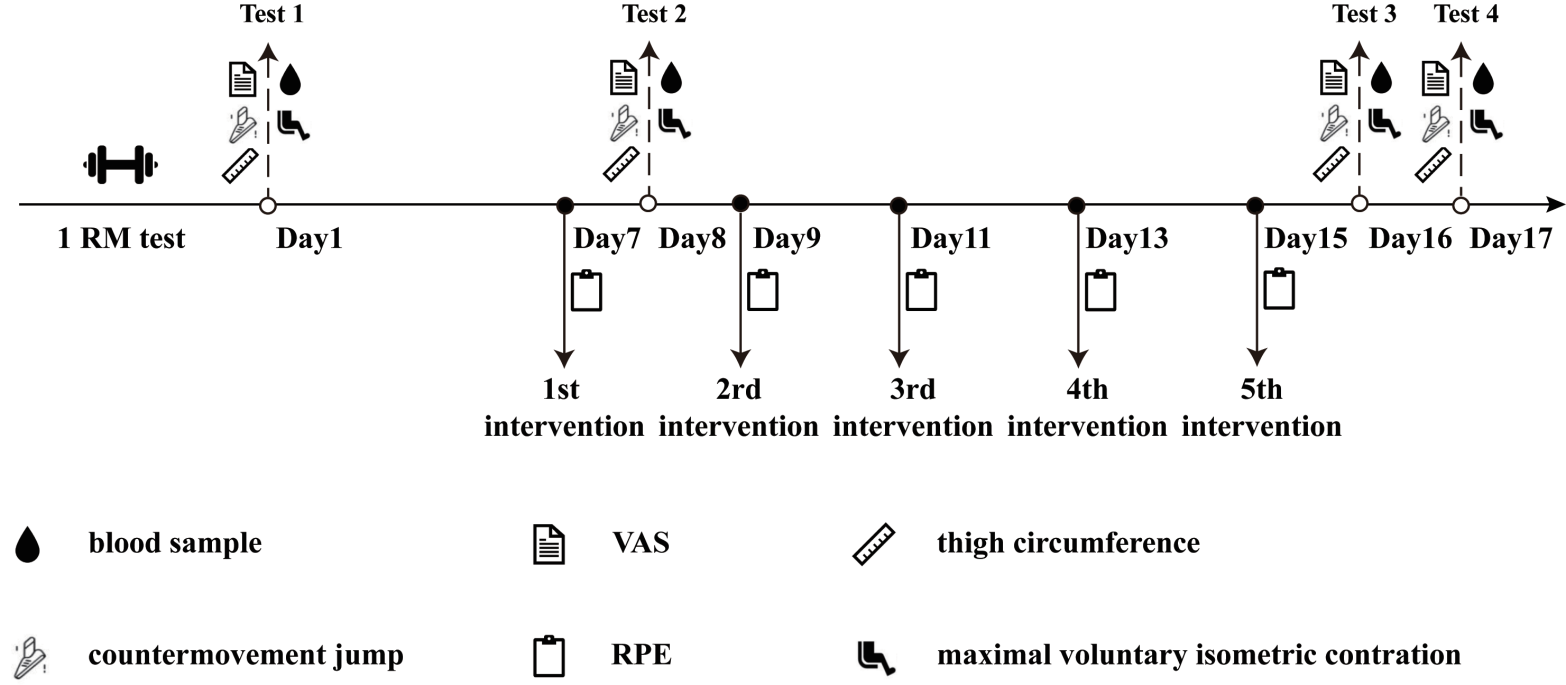
Study design.

Thirty-seven participants were familiarized with the protocol and then randomized to one of four groups: CON, TRA, IPC, or PEIC. The PEIC group comprised 10 participants, while the remaining groups each had 9 participants. The CON group did not receive any additional treatment before or after each ST session. The TRA group underwent static stretching and foam rolling following each ST session. The IPC group received IC 30 minutes before each ST session, while PEIC received IC immediately after each ST session. Strength training sessions were conducted on alternate days, amounting to a total of five sessions. Measurements of muscle soreness, thigh circumference, CMJ, knee isokinetic muscle strength (PT and RPT), and serum markers including CK, LDH, T-AOC, SOD, MDA, CRP, and IL-6 were taken at multiple time points: baseline, 24 h after the first session (1^st^–24 h), and 24 and 48 h after the final session (5^th^–24 h, 5^th^–48 h) (Fig. 1).

### Familiarization and 1RM test

During the familiarization trial, participants were exposed to the IC protocol. The 1RM squat test was also performed to determine the initial load for ST. The procedures used for the 1RM back squat tests were as follows. Participants completed 2 warm-up sets: 10 repetitions using approximately 40% of their estimated 1RM followed by 5 repetitions using approximately 60% of their estimated 1RM. The load was then increased to approximately 85% of the estimated 1RM, and after 5 min rest, 1 repetition was performed. 5%–10% weight was then added for each subsequent 1 repetition set, until the participant was no longer able to complete a repetition correctly. The 1RM was determined as the last weight used in which the participant successfully completed the lift with proper form through the entire range of motion.

### Strength training protocol

To induce muscle fatigue, participants engaged in a rigorous workout consisting of 6 sets of 6 repetitions of the weighted squat at 80% 1RM, with a 2-minute rest period between sets. Additionally, participants performed 20 self-weighted squat jump-ups, organized into 5 sets with 2-minute intervals between sets. Participants were consistently provided with verbal encouragement throughout the exercise protocol to ensure maximal effort and compliance.

### Intervention procedure

**FIG. 2:**
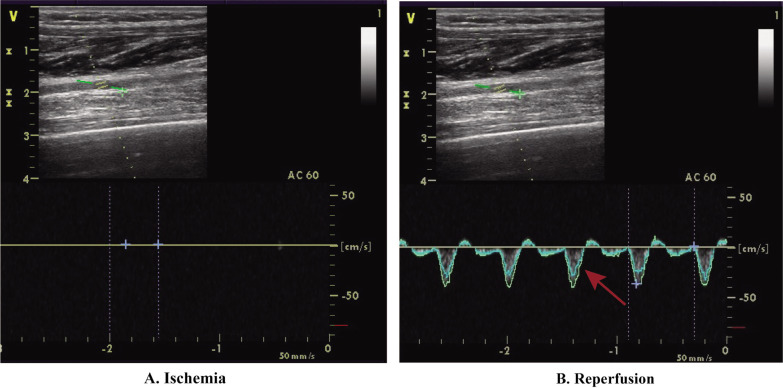
Doppler ultrasonography.

The CON group, serving as the control, received no extra interventions. The TRA group engaged in static stretching and foam rolling. Static stretching targeted the gluteus maximus, quadriceps, and hamstrings on both sides of the body. Each muscle was stretched for 45 seconds, followed by a 15-second rest, and this cycle was repeated three times, resulting in an 18-minute session. After 3 minutes, bilateral foam rolling was simultaneously applied to the gluteus maximus, quadriceps, and hamstrings. Each muscle was massaged for 45 seconds, then rested for 15 seconds, and this was repeated three times for a total of 9 minutes. The total duration of TRA was 30 minutes. In IPC, the ischemic protocol was administered before each ST session, while PEIC underwent the ischemic protocol after each ST. The Doppler ultrasound (LOGIQ E9, General Electric, Boston) was used to closely monitor the IC procedure and arterial inflow to ensure complete closure of the arterial inflow (Fig. 2). All ultrasound procedures were conducted by a physician trained in ultrasound imaging. During the ischemic protocol, participants were lying in a supine position, and non-elastic blood pressure cuffs (Yuwell, China, width: 14 cm) were bilaterally positioned [7] under the gluteal line upper thighs. These cuffs were rapidly inflated to 220 mmHg for 5 minutes to impede arterial inflow. This process was repeated three times, with each compression episode followed by 5 minutes of reperfusion period (cuff release). The intervention protocol, validated for complete occlusion of vascular arterial inflow [17] and induction of physiological responses [18], had a total duration of 30 minutes.

### Perceptual measures

After each intervention, participants were asked to report their rate of perceived exertion (RPE) using the Borg 6–20 RPE scale. Muscle soreness was assessed using a visual analog scale (VAS). The scale ranged from 0 (no pain) to 10 (extreme pain). Participants assumed the anatomical position with their hands on their hips and were instructed to hold a half-squat position with a 90 knee angle. They were then asked to indicate their level of perceived soreness on the provided scale.

### Blood samples

Blood collection occurred four times: baseline, 1^st^–24 h, 5^th^–24 h, and 5^th^–48 h. Approximately 5 mL of venous blood was collected from the antecubital vein and immediately placed in a refrigerated centrifuge. It was then spun at 3500 rev/min for 10 minutes at 4. The sample was stored at -80 for later analysis. Serum MDA was determined using a commercially available kit (Nanjing Jiancheng Bioengineering Institute, Nanjing, China) based on thiobarbituric acid (TBA) reactivity. Serum SOD activity was assessed using a commercially available kit (Nanjing Jiancheng Bioengineering Institute, Nanjing, China) based on the auto-oxidation of hydroxylamine. Serum T-AOC was detected by a commercially available kit (Nanjing Jiancheng Bioengineering Institute, Nanjing, China) with the ABTS method. The serum CRP and IL-6 levels were determined by enzymelinked immunosorbent assays (ELISAs) (JL, Jianglai Biological, Shanghai, China). Serum CK and LDH were analyzed using an automatic biochemistry analyzer (UniCel DxC 600 Synchron, Beckman Coulter, USA).

### Thigh circumference

Thigh circumference was measured on the bilateral legs, midway between the greater trochanter and the lateral epicondyle of the femur. An anthropometric tape was used to measure the thigh circumference in an anatomical position. The site of measurement was marked using a semi-permanent pen and performed by the same experienced researcher. This was done to ensure consistent measurements between test days.

### Countermovement jump

The lower limb muscular power was assessed by performing each CMJ with hands on hips. Following a standardized warm-up consisting of 5 incremental sub-maximal CMJ efforts, participants performed 3 maximal CMJ efforts with 60 seconds of recovery between each attempt. The participants stood on a portable electronic mat (GMCS. ZT, Canada) and dropped to a self-selected level before performing a maximal jump. The highest value achieved was recorded.

### Knee isokinetic muscle strength

Knee isokinetic muscle strength was measured by an isokinetic muscle strength testing system (Biodex System 4, USA). Participants sat in standardized position, arms crossed, hips and knees flexed 90°. The position was measured before each contraction using a goniometer to minimize error. To prevent unwanted movement, straps were placed across the torso and hips. Before the formal examination, participants performed exercise at submaximal intensity 5 times, followed by a 60-second resting period. During the formal test, participants were required to extend their keens at a velocity of 60°/s for 5 repetitions at maximum resistance. The procedure was executed in a specific order beginning with the right leg and concluding with the left.

### Statistics Analysis

The basic characteristics among the four groups were analyzed by one-way ANOVA. A repeated two-way ANOVA (Treatment, 4 × Time, 4) was used to analyze differences in variables measured between conditions and trials, with treatment as the between-subject factor and time as the within-subjects factor. Effect sizes (ES) for main effects and interactions were determined by partial eta squared (η2). Partial eta squared values were classified as small (0.01–0.059), moderate (0.06–0.137) and large (> 0.137). A Bonferroni post-hoc pair-wise comparison was performed after significant interactions. A Greenhouse-Geisser correction was applied due to the violation of the assumption of sphericity. For data that do not follow a normal distribution (VAS, RPE), the Kruskal-Wallis test was employed to analyze differences between groups at the same time point, with Dunn’s test used for post-hoc analysis. For pairwise comparisons, effect sizes (ES) were determined by Cohen’s d which was characterized as large (d > 0.8), moderate (d between 0.8 and 0.5), small (d between 0.49 and 0.20), and trivial (d < 0.2). The statistical analyses were performed using IBM SPSS 27 (SPSS, Chicago, IL). The data is presented as mean ± SD. Statistical significance was set a priori at *P* < 0.05.

## RESULTS

### Perceptual measures

After each training session, the RPE in the TRA group was significantly lower than in the CON, IPC, and PEIC groups (all *P* < 0.05) (Table 2). There was no significant difference in VAS among the groups at Baseline, 1^st^–24 h, 5^th^–24 h, and 5^th^–48 h (all *P* > 0.05) (Table 2).

**TABLE 2:** Change in RPE, VAS and bilateral thigh circumference.

	Timepoint	CON	TRA	IPC	PEIC
RPE	1^st^ intervention	15 ± 1^*^	10 ± 3	17 ± 2^*^	13 ± 1^*^
	2^rd^ intervention	13 ± 2^*^	10 ± 4	13± 3^*^	13 ± 3^*^
	3^rd^ intervention	12 ± 0^*^	8 ± 2	14 ± 2^*^	12 ± 2^*^
	4^th^ intervention	12 ± 0^*^	8 ± 1	14 ± 1^*^	12 ± 2^*^
	5^th^ intervention	12 ± 2^*^	9 ± 1	14 ± 1^*^	12 ± 2^*^

VAS	Baseline	0	0	0	0
	1^st^–24 h	6 ± 1	4 ± 3	5 ± 2	8 ± 2
	5^th^–24 h	2 ± 2	1 ± 1	2 ± 2	2 ± 2
	5^th^–48 h	1 ± 1	1 ± 1	1 ± 1	2 ± 2

Left TC (cm)	Baseline	53.9 ± 2.2	54.0 ± 2.3	55.0 ± 3.7	56.1 ± 3.1
	1^st^–24 h	56.4 ± 2.7	54.5 ± 1.0	56.0 ± 3.1	57.6 ± 3.1
	5^th^–24 h	56.4 ± 3.6	54.4 ± 1.6	56.3 ± 3.8	56.3 ± 3.8
	5^th^–48 h	56.5 ± 3.4	54.2 ± 4.1	56.1 ± 3.3	56.1 ± 3.3

Right TC (cm)	Baseline	54.2 ± 2.1	53.6 ± 1.1	55.1 ± 3.9	55.3 ± 4.7
	1^st^–24 h	56.6 ± 2.7	55.5 ± 2.1	56.3 ± 2.6	57.6 ± 3.0
	5^th^–24 h	56.7 ± 3.5	55.6 ± 2.1	56.8 ± 3.3	57.9 ± 2.8
	5^th^–48 h	56.3 ± 2.6	55.4 ± 1.0	56.7 ± 3.5	57.2 ± 3.5

* Significantly different from TRA (*P* < 0.05). Values are presented as mean SD.

### Thigh circumference

There was a significant effect of time on left TC (*P* < 0.001, η^2^ = 0.17) and right TC (*P* < 0.001, η^2^ = 0.37) but no interaction between time and group (all *P* > 0.05) (Table 2). The baseline values of bilateral TC were significantly lower than at 1^st^–24 h, 5^th^–24 h, and 5^th^–48 h.

### CMJ height

**FIG. 3:**
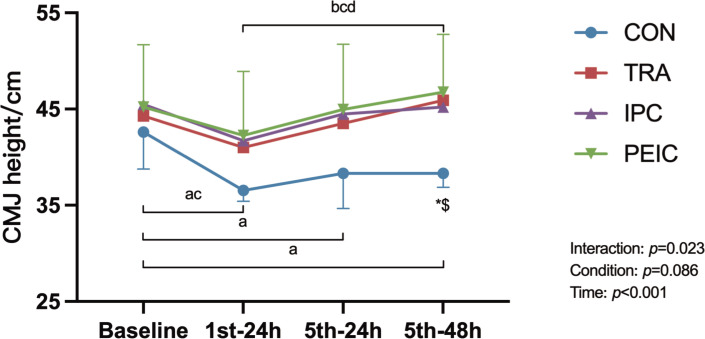
Change in the countermovement jump (CMJ). Note: ^a^ significant difference within the CON group (*P* <0.05), ^b^ significant difference within the TRA group (*P* <0.05), ^c^ significant difference within the IPC group (*P* <0.05), ^d^ significant difference within the PEIC group (*P* <0.05), *TRA group significantly different from CON group (*P* <0.05), ^#^IPC group significantly different from CON group (*P* <0.05), ^$^PEIC group significantly different from CON group (P<0.05).

Figure 3 shows the change in CMJ height. Baseline CMJ height had no differences between groups (*P* > 0.05). After ST, values decreased in CON at 1^st^–24 h (*P* < 0.001, Cohen’s d = 2.15, 95% CI [0.89, 3.40]), 5^th^–24 h (*P* = 0.002, Cohen’s d = 1.14, 95% CI [0.06, 2.22]), and 5^th^–48 h (*P* < 0.001, Cohen’s d = 1.48, 95% CI [0.35, 2.61]) compared to baseline. In IPC, values decreased at 1^st^–24 h (*P* = 0.020, Cohen’s d = 0.48, 95% CI [0.45, 1.49]) only. Notably, in TRA and PEIC, values remained unchanged at all time points compared to the baseline. Furthermore, TRA and PEIC were higher compared to CON at 5^th^–48 h (*P* = 0.037, Cohen’s d = -2.74, 95% CI [-4.13, -1.35]; *P* = 0.013, Cohen’s d = -1.88, 95% CI [-3.05, -0.72]).

### Knee isokinetic strength

**FIG. 4:**
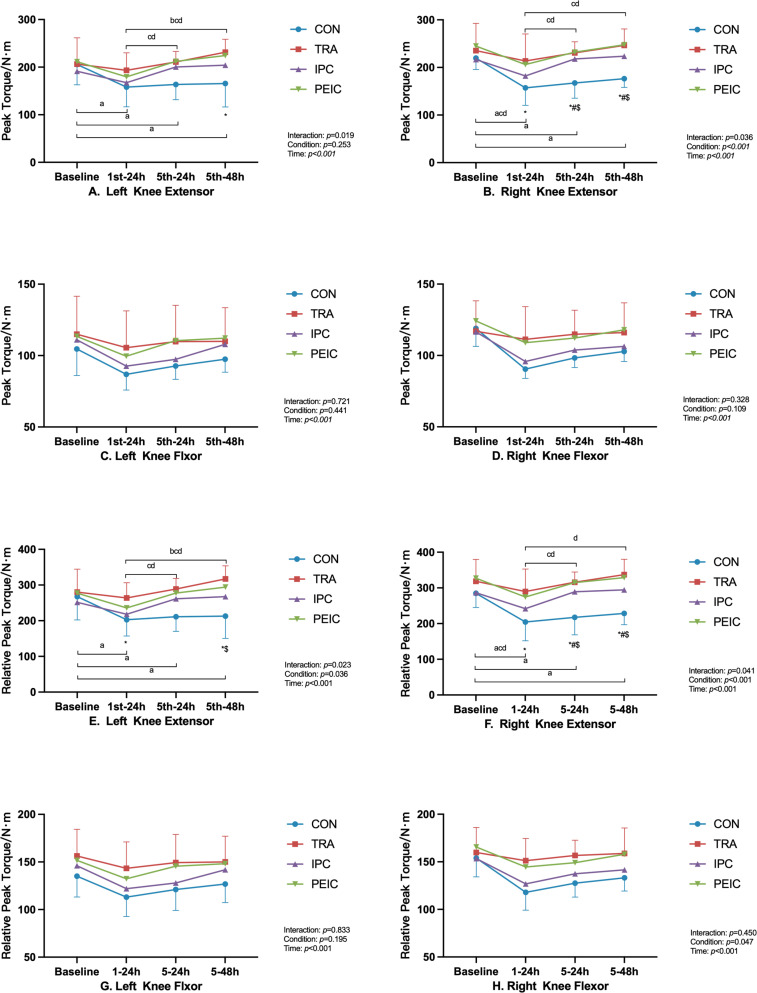
Change in knee isometric muscle strength. Note: ^a^ significant difference within the CON group (*P* <0.05), ^b^ significant difference within the TRA group (*P* <0.05), ^c^ significant difference within the IPC group (*P* <0.05), ^d^ significant difference within the PEIC group (*P* <0.05), *TRA group significantly different from CON group (*P* <0.05), ^#^IPC group significantly different from CON group (*P* <0.05), ^$^PEIC group significantly different from CON group (*P* <0.05).

Figure 4 shows the change in peak torque (PT) and relative peak torque (RPT). Left quadriceps PT had no differences between groups at baseline (*P* > 0.05). After ST, values decreased in CON at 1^st^–24 h (*P* = 0.010, Cohen’s d = 1.12, 95% CI [0.05, 2.20]), 5^th^–24 h (*P* = 0.020, Cohen’s d = 1.11, 95% CI [0.04, 2.19]), and 5^th^–48 h (*P* = 0.038, Cohen’s d = 0.87, 95% CI [0.18, 1.91]) compared to baseline, whereas in TRA, IPC, and PEIC, values remained unchanged at all time points compared to baseline. Additionally, in IPC and PEIC, the left quadriceps PT at 1^st^–24 h was lower than the 5^th^–24 h (*P* = 0.036, Cohen’s d = -0.61, 95% CI [-1.63, -0.41]; *P* = 0.034, Cohen’s d = -0.51, 95% CI [-1.47, -0.44]). Furthermore, TRA was higher than CON at 5^th^–48 h (*P* = 0.043, Cohen’s d = -1.66, 95% CI [-2.81, -0.50]).

Baseline Right quadriceps PT had no differences between groups (*P* > 0.05). After ST, values decreased in CON at 1^st^–24 h (*P* < 0.001, Cohen’s d = 2.02, 95% CI [0.79, 3.25]), 5^th^–24 h (*P* < 0.001, Cohen’s d = 1.85, 95% CI [0.65, 3.04]), and 5^th^–48 h (*P* = 0.004, Cohen’s d = 2.03, 95% CI [0.8, 3.27]) compared to baseline. However, in IPC and PEIC, values decreased at 1^st^–24 h (*P* = 0.038, Cohen’s d = 0.38, 95% CI [0.26, 1.39]; *P* = 0.009, Cohen’s d = 1.81, 95% CI [0.62, 2.99]) only, while in TRA, values remained unchanged at all time points compared to the baseline. Notably, in IPC and PEIC, values at 1^st^–24 h were lower than the 5^th^–24 h (*P* = 0.015, Cohen’s d = -1.56, 95% CI [-2.70, -0.42]; *P* = 0.032, Cohen’s d = -0.65, 95% CI [-1.61, -0.31]). Furthermore, TRA was higher compared to CON at 1^st^–24 h (*P* = 0.040, Cohen’s d = -1.18, 95% CI [-2.26, -0.10]). Additionally, TRA, IPC, and PEIC were higher than CON at 5^th^–24 h (*P* < 0.001, Cohen’s d = -2.27, 95% CI [-3.55,-0.99]; *P* = 0.007, Cohen’s d = -1.73, 95% CI [-2.91, -0.56]; *P* < 0.001, Cohen’s d = -1.89, 95% CI [-3.06, -0.73]) and 5^th^–48 h (*P* < 0.001, Cohen’s d = -2.54, 95% CI [-3.89, -1.20]; *P* = 0.025, Cohen’s d = -1.64, 95% CI [-2.80, -0.49]; *P* < 0.001 Cohen’s d = -2.39, 95% CI [-3.66, -1.12]).

For bilateral knee flexors PT, there was an effect of time (all *P* < 0.05) but no interaction between time and group (all *P* > 0.05).

Baseline RPT of the left quadriceps had no differences between groups (*P* > 0.05). After ST, values were decreased in CON at 1^st^–24 h (*P* = 0.009, Cohen’s d = 1.15, 95% CI [0.07, 2.22]), 5^th^–24 h (*P* = 0.017, Cohen’s d = 1.04, 95% CI [0.03, 2.10]), and 5^th^–48 h (*P* = 0.038, Cohen’s d = 0.86, 95% CI [0.19, 1.90]) compared to baseline, whereas in TRA, IPC, and PEIC, values remained unchanged at all time points compared to baseline. Additionally, in IPC and PEIC, values at 1^st^–24 h were lower than the 5^th^–24 h (*P* = 0.048, Cohen’s d = -0.97, 95% CI [-2.03, -0.08]; *P* = 0.046, Cohen’s d = -0.57, 95% CI [-1.53, -0.39]). Furthermore, TRA was higher than CON at 5^th^–24 h (*P* = 0.018, Cohen’s d = -2.19, 95% CI [-3.45, -0.92]). Additionally, TRA and PEIC were higher compared to CON at 5^th^–48 h (*P* = 0.003, Cohen’s d = -2.05, 95% CI [-3.28, -0.81]; *P* = 0.022, Cohen’s d = -1.15, 95% CI [-2.20, -0.11]).

Baseline RPT of the right quadriceps had no differences between groups (*P* > 0.05). After ST, values decreased in CON at 1^st^–24 h (*P* < 0.001, Cohen’s d = 1.73, 95% CI [0.56, 2.90]), 5^th^–24 h (*P* < 0.001, Cohen’s d = 1.51, 95% CI [0.38, 2.64]), and 5^th^–48 h (*P* = 0.006, Cohen’s d = 1.57, 95% CI [0.42, 2.71]) compared to baseline. However, in IPC and PEIC, values were decreased at 1^st^–24 h (*P* = 0.040, Cohen’s d = 1.63, 95% CI [0.48, 2.78]; *P* = 0.006) Cohen’s d = 0.83, 95% CI [0.15, 1.81] only, while in TRA, values remained unchanged at all time points compared to baseline. Notably, in IPC and PEIC, values at 1^st^–24 h were lower than the 5^th^–24 h (*P* = 0.014, Cohen’s d = -1.34, 95% CI [-2.44, -2.03]; *P* = 0.036, Cohen’s d = -0.67, 95% CI [-1.63, -0.30]). Furthermore, TRA was higher compared to CON at 1^st^–24 h (*P* = 0.015, Cohen’s d = -1.47, 95% CI [-2.59, -0.34]). Additionally, TRA, IPC, and PEIC were higher than CON at 5^th^–24 h (*P* < 0.001, Cohen’s d = -2.45, 95% CI [-3.78, -1.13]; *P* = 0.009, Cohen’s d = -1.61, 95% CI [-2.76, -0.46]; *P* < 0.001, Cohen’s d = -1.88, 95% CI [-3.04, -0.71]) and 5^th^–48 h (*P* < 0.001, Cohen’s d = -2.87, 95% CI [-4.29, -1.44]; *P* = 0.019, Cohen’s d = -1.89, 95% CI [-3.10, -0.69]; *P* < 0.001, Cohen’s d = -2.13, 95% CI [-3.34, -0.92]).

For RPT of bilateral knee flexors, there was an effect of time (all *P* < 0.05) but no interaction between time and group (all *P* > 0.05).

### Muscle damage biomarkers

**FIG. 5:**
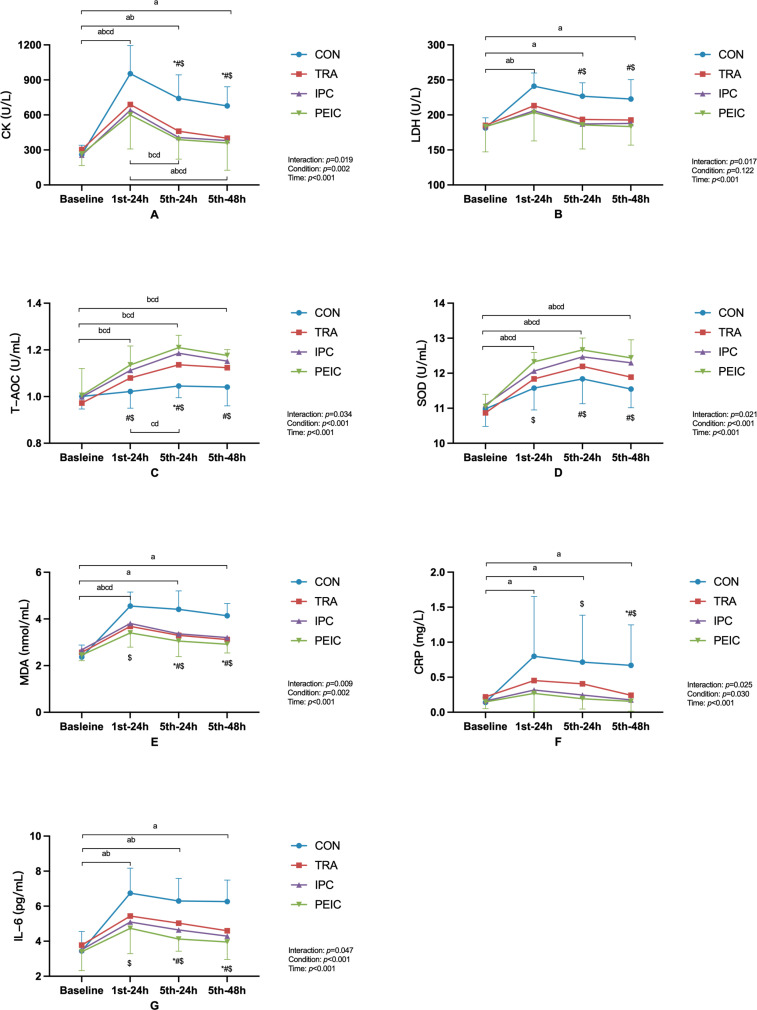
Change in serum biomarkers. Note: ^a^ significant difference within the CON group (*P* <0.05), ^b^ significant difference within the TRA group (*P* <0.05), ^c^ significant difference within the IPC group (*P* <0.05), ^d^ significant difference within the PEIC group (*P* <0.05); *TRA group significantly different from CON group (*P* <0.05), ^#^IPC group significantly different from CON group (*P* <0.05), ^$^PEIC group significantly different from CON group (*P* <0.05).

Figure 5 (A and B) shows the change in CK and LDH. Baseline CK had no differences between groups (*P* > 0.05). After ST, CK increased in CON at 1^st^–24 h (*P* < 0.001, Cohen’s d = -3.86, 95% CI [-5.55, -2.17]), 5^th^–24 h (*P* < 0.001, Cohen’s d = -3.13, 95% CI [-4.62, -1.64]), and 5^th^–48 h (*P* < 0.001, Cohen’s d = -3.23, 95% CI [-4.75, -1.72]) compared to baseline. However, in TRA, values increased at 1^st^–24 h (*P* = 0.002, Cohen’s d = -2.24, 95% CI [-3.51, -0.96]) and 5^th^–24 h (*P* = 0.004, Cohen’s d = -1.30, 95% CI [-2.40, -0.20]) only, whereas in IPC and PEIC, an increase was observed at 1^st^–24 h (*P* = 0.002, Cohen’s d = -1.36, 95% CI [-2.47, -0.25]; *P* = 0.005, Cohen’s d = -1.53, 95% CI [-2.60, -0.46]) only. Notably, in TRA, IPC, and PEIC, values at 1^st^–24 h were higher than 5^th^–24 h (*P* = 0.033, Cohen’s d = 1.43, 95% CI [0.31, 2.55]; *P* = 0.028, Cohen’s d = 0.76, 95% CI [0.27, 1.80]; *P* = 0.038, Cohen’s d = 0.89, 95% CI [0.09, 1.88]). Furthermore, TRA, IPC, and PEIC were lower compared to CON at 5^th^–24 h (*P* = 0.006, Cohen’s d = 1.76, 95% CI [0.58, 2.93]; *P* < 0.001, Cohen’s d = 1.78, 95% CI [0.60, 2.96]; *P <* 0.001, Cohen’s d = 1.91, 95% CI [0.74, 3.08]) and 5^th^–48 h (*P* = 0.007, Cohen’s d = 2.15, 95% CI [0.89, 3.40]; *P* = 0.004, Cohen’s d = 1.96, 95% CI [0.74, 3.17]; *P* = 0.001, Cohen’s d = 1.56, 95% CI [0.45, 2.67]).

Baseline LDH had no differences between groups (*P* > 0.05). After ST, LDH increased in CON at 1^st^–24 h (*P* < 0.001, Cohen’s d = -3.57, 95% CI [-5.18, -1.96]), 5^th^–24 h (*P* < 0.001, Cohen’s d = -2.67, 95% CI [-4.05, -1.30]), and 5^th^–48 h (*P* < 0.001, Cohen’s d = -1.87, 95% CI [-3.07, -0.67]) compared to baseline. However, values increased in TRA at 1^st^–24 h (*P* = 0.018, Cohen’s d = -0.57, 95% CI [-1.59, -0.45]) only, while in IPC and PEIC, values remained unchanged at all time points compared to the baseline. Furthermore, lower values were observed in IPC and PEIC compared to the CON group and 5^th^–24 h (*P* = 0.025, Cohen’s d = 1.51, 95% CI [0.38, 2.65]; *P* = 0.016, Cohen’s d = 1.45, 95% CI [0.36, 2.54]) and 5^th^–48 h (*P* = 0.036, Cohen’s d = 1.61, 95% CI [0.46, 2.76]; *P* = 0.010, Cohen’s d = 1.46, 95% CI [0.37, 2.55]).

### Oxidative stress biomarkers

Figure 5 (C, D, and E) shows the change in T-AOC, SOD, and MDA. Baseline T-AOC had no differences between groups (*P* > 0.05). After ST, T-AOC remained unchanged at all time points compared to the baseline in CON, whereas T-AOC in TRA, IPC, and PEIC increased at 1^st^–24 h (*P* = 0.020, Cohen’s d = -2.05, 95% CI [-3.29, -0.82]; *P* = 0.012, Cohen’s d = -1.27, 95% CI [-2.36, -0.17]; *P* = 0.002, Cohen’s d = -1.31, 95% CI [-2.35, -0.28]), 5^th^–24 h (*P* < 0.001, Cohen’s d = -2.79, 95% CI [-4.20, -1.39]; *P* < 0.001, Cohen’s d = -2.28, 95% CI [-3.56, -1.00] *P* < 0.001, Cohen’s d = -2.28, 95% CI [-3.49, -1.07]), and 5^th^–48 h (*P* = 0.001, Cohen’s d = -1.82, 95% CI [-3.00, -0.63]; *P* = 0.001, Cohen’s d = -1.69, 95% CI [-2.85, -0.53]; *P* < 0.001, Cohen’s d = -2.05, 95% CI [-3.21, -0.89]) compared to baseline. Notably, in IPC and PEIC, values at 1^st^–24 h were lower than 5^th^–24 h (*P* = 0.033, Cohen’s d = -1.23, 95% CI [-2.32, = 0.14]; *P* = 0.021, Cohen’s d = -1.08, 95% CI [-2.08, -0.07]). Furthermore, higher values were observed in IPC and PEIC compared to CON at 1^st^–24 h (*P* = 0.040, Cohen’s d = -1.28, 95% CI [-2.38, -0.19]; *P* = 0.004, Cohen’s d = -1.49, 95% CI [-2.59, -0.39]) and 5^th^–48 h (*P* = 0.017, Cohen’s d = -1.44, 95% CI [-2.57, -0.32]; *P* = 0.002, Cohen’s d = -2.31, 95% CI [-3.57, -1.06]). Additionally, TRA, IPC, and PEIC were higher than CON at 5^th^–24 h (*P* = 0.003, Cohen’s d = -1.85, 95% CI [-3.05, -0.66]; *P* < 0.001, Cohen’s d = -2.87, 95% CI [-4.29, -1.44]; *P* < 0.001, Cohen’s d = -3.19, 95% CI [-4.65, -1.73]).

Baseline SOD had no differences between groups (*P* > 0.05). After ST, SOD increased at all time points in four groups (*P* < 0.001) compared to baseline. Furthermore, higher values were observed in PEIC compared to CON at 1^st^–24 h (*P* = 0.005, Cohen’s d = -1.31, 95% CI [-2.38, -0.24]). Additionally, IPC and PEIC were higher than CON at 5^th^–24 h (*P* = 0.025, Cohen’s d = -0.89, 95% CI [-1.94, 0.16] *P* = 0.001, Cohen’s d = -1.61, 95% CI [-2.73, -0.50]) and 5^th^–48 h (*P* = 0.003, Cohen’s d = -0.78, 95% CI [-1.82, 0.26]; *P* < 0.001, Cohen’s d = -1.13, 95% CI [-2.17, -0.08]).

Baseline MDA had no differences between groups (*P* > 0.05). After ST, MDA was increased in CON at 1^st^–24 h (*P* < 0.001, Cohen’s d = -3.92, 95% CI [-5.63, -2.21]), 5^th^–24 h (*P* < 0.001, Cohen’s d = -3.07, 95% CI [-4.54, -1.59]), and 5^th^–48 h (*P* < 0.001, Cohen’s d = -3.41, 95% CI [-4.97, -1.84]) compared to baseline, whereas MDA in TRA, IPC, and PEIC increased at 1^st^–24 h (*P* = 0.012, Cohen’s d = -1.32, 95% CI [-2.42, -0.22]; *P* = 0.010, Cohen’s d = -1.19, 95% CI [-2.28, -0.11]; *P* = 0.029, Cohen’s d = -2.05, 95% CI [-3.21, -0.89]) only. Furthermore, lower values were observed in PEIC compared to CON at 1^st^–24 h (*P* = 0.040, Cohen’s d = 1.91, 95% CI [0.74, 3.08]). Additionally, TRA, IPC, and PEIC were lower than CON at 5^th^–24 h (*P* = 0.022, Cohen’s d = 1.41, 95% CI [0.29, 2.53] *P* = 0.035, Cohen’s d = 1.33, 95% CI [0.23, 2.44]; *P* = 0.003, Cohen’s d = 1.87, 95% CI [0.71, 3.04]) and 5^th^–48 h (*P* = 0.005, Cohen’s d = 1.75, 95% CI [0.58, 2.93] *P* = 0.011, Cohen’s d = 1.43, 95% CI [0.31, 2.55]; *P* < 0.001, Cohen’s d = 2.69, 95% CI [1.35, 4.03]).

### Inflammation biomarkers

Figure 5 (F and G) shows the change in CRP and IL-6. Baseline CRP had no differences between groups (*P* > 0.05). After ST, CRP increased in CON at 1^st^–24 h (*P* = 0.001, Cohen’s d = -1.09, 95% CI [-2.61, -0.02]), 5^th^–24 h (*P* < 0.001, Cohen’s d = -1.21, 95% CI [-2.29, -0.12]), and 5^th^–48 h (*P* < 0.001, Cohen’s d = -1.28, 95% CI [-2.38, -0.18]) compared to baseline, whereas CRP in TRA, IPC, and PEIC remained unchanged at all time points compared to the baseline. Furthermore, lower values were observed in PEIC compared to CON at 5^th^–24 h (*P* = 0.027, Cohen’s d = 1.11, 95% CI [0.07, 2.15]). Additionally, TRA, IPC, and PEIC were lower than CON at 5^th^–48 h (*P* = 0.034, Cohen’s d = 1.01, 95% CI [0.05, 2.07]; *P* = 0.010, Cohen’s d = 1.19, 95% CI [0.11, 2.28]; *P* = 0.005, Cohen’s d = 1.25, 95% CI [0.19, 2.31]).

Baseline IL-6 had no differences between groups (*P* > 0.05). After ST, IL-6 was increased in CON at 1^st^–24 h (*P* < 0.001, Cohen’s d = -2.58, 95% CI [-3.94, -1.23]), 5^th^–24 h (*P* < 0.001, Cohen’s d = -2.37, 95% CI [-3.67, -1.07]), and 5^th^–48 h (*P* < 0.001, Cohen’s d = -2.40, 95% CI [-3.71, -1.09]) compared to baseline. However, IL-6 increased in TRA at 1^st^–24 h (*P* = 0.039, Cohen’s d = -1.30, 95% CI [-2.40, -0.20]) and 5^th^–24 h (*P* = 0.038, Cohen’s d = -1.56, 95% CI [-2.70, -0.42]) only, while in IPC and PEIC, it remained unchanged at all time points compared to the baseline. Furthermore, lower values were observed in PEIC compared to CON at 1^st^–24 h (*P* = 0.023, Cohen’s d = 1.40, 95% CI [0.32, 2.48]). Additionally, TRA, IPC, and PEIC were lower than CON at 5^th^–24 h (*P* = 0.044, Cohen’s d = 1.33, 95% CI [0.23, 2.44]; *P* = 0.004, Cohen’s d = 1.37, 95% CI [0.26, 2.48]; *P* < 0.001, Cohen’s d = 2.13, 95% CI [0.92, 3.35]) and 5^th^–48 h (*P* = 0.006, Cohen’s d = 1.46, 95% CI [0.33, 2.58]; *P* < 0.001, Cohen’s d = 2.11, 95% CI [0.86, 3.35]; *P* < 0.001, Cohen’s d = 2.07, 95% CI [0.87, 3.27]).

## DISCUSSION

The primary objective of this study was to investigate the effects of using IC before or after ST on functional aspects, muscle damage, inflammatory activity, and oxidative stress. In addition, a comparison was made between IC and traditional recovery methods (static stretching combined with foam rolling). The findings of this study suggested that both IPC and PEIC had the potential to reduce muscle damage, alleviate oxidative stress, attenuate inflammatory responses, enhance antioxidant capacity, and maintain lower extremity strength performance when compared to CON. Furthermore, the results indicated that repeated use of IC significantly reduced muscle damage, enhanced antioxidant capacity, and improved lower extremity strength performance compared to acute administration. Moreover, PEIC led to a faster recovery of CMJ than IPC during acute administration. However, after five repetitions, IPC and PEIC had similar effects. In comparison to IC, the results indicated that the acute use of TRA led to a faster recovery of lower extremity strength. In contrast, IC led to a faster recovery of muscle damage and inflammation when used both acutely and repeatedly.

The exercise protocol used in our study was complex training, combining traditional resistance training and rapid stretch load training. The training protocol was more consistent with the actual training of the participants in terms of intensity and modality considerations. The results of our study indicated that the ST protocol was effective in inducing muscle fatigue, as objective measures (such as CK) showed muscle fatigue post-ST. Therefore, the results of the observed effects of TRA, IPC, and PEIC on recovery were reliable.

Recent studies have shown that IPC decreased biomarkers related to muscle damage [7] and inflammatory processes [19], while also attenuating exercise-induced oxidative stress [20]. Evidence also suggested IPC and PEIC could accelerate post-exercise recovery [7, 8, 10–13, 19, 21]. These beneficial results were observed in various exercise modalities and protocols, such as eccentric contractions [7, 10, 12], marathon running [19, 20], cycling [11] and simulated soccer physical demand test [21]. The present study’s findings aligned with and reinforced this existing body of evidence. Our study explored the impact of IC on key biomarkers, including CK, LDH, T-AOC, SOD, MDA, IL-6, and CRP. CK and LDH are reliable indicators of muscle damage, T-AOC and SOD reflect the body’s antioxidant defense mechanisms, MDA is a marker of oxidative stress, and IL-6 and CRP are critical indicators of post-exercise inflammation. Our findings indicated that in the IPC and PEIC groups, CK and MDA levels returned to baseline at 5^th^–24 h. LDH, IL-6, and CRP levels showed no significant differences compared to baseline at any time points, while T-AOC and SOD levels significantly increased following the interventions. Observations in our study indicated that both IPC and PEIC were effective in modulating muscle damage, inflammation, and oxidative stress following ST. These findings hold practical significance for individuals who are seeking approaches to speed recovery from fatiguing exercise. However, for elite athletes, further investigation is needed.

To date, no study has investigated the differences between IPC and PEIC. The findings from this study showed that the application of IC before or after ST had a positive effect on the recovery of ST. This was aligned with the previous investigation that PEIC attenuated the decrease of CMJ at 24 hours [21]. Interestingly, acute IC applied after ST had a more pronounced effect on maintaining CMJ height compared to its application before ST. This might be due to IPC primarily enhancing performance rather than aiding recovery from fatigue during acute applications. Besides, the interaction between the timing of IC application and exercise could have influenced the effects of IPC and PEIC. In contrast, repeated application of IC, either before or after each ST appeared to produce a similar effect. Actually, IPC and PEIC were relative concepts in the context of training. PEIC served as a post-conditioning strategy for the previous training session or day, as well as a pre-conditioning approach for the following training session or day. It’s important to note that PEIC was applied earlier in the training schedule and at longer intervals from the start of training than IPC. In summary, when administered acutely, individuals who chose PEIC over IPC would have better positive results. In contrast, with five repeated applications, our results suggested that IC could be applied before or after ST depending on individual preference.

This study also shed light on the differences between IC and traditional recovery methods. Static stretching and foam rolling are the most commonly used recovery methods [22–24]. However, no studies have compared IC with static stretching and foam rolling. Interestingly, our findings indicated that in the TRA group, right extensor RPT and PT returned to baseline at 1^st^–24 h, while in the IPC and PEIC groups, right extensor RPT and PT remained significantly lower than baseline. However, LDH and IL-6 levels in the TRA group were significantly higher than baseline, whereas in the IPC and PEIC groups, LDH and IL-6 levels returned to baseline at 1^st^–24 h. These data suggested that acute TRA led to a faster recovery of lower extremity strength compared to IC, while IC led to a faster recovery of muscle damage and inflammation than TRA. Our study found that TRA had the lowest RPE at all the time points compared to other groups (Table 2). Reduced muscle soreness and improved perceptions of recovery could significantly impact athletes’ physical performance and, consequently, athletic performance [25, 26]. Additionally, Rahami et al. [27] found that foam rolling could accelerate the recovery of psychological characteristics. Thus, static stretching and foam rolling might provide a relatively comfortable sensation that could have positive psychological effects on participants, thereby contributing to an accelerated recovery from external load following ST. IC might engage signaling pathways and effects that differed from static stretching and foam rolling, potentially emphasizing adaptive cellular changes. For instance, IC had been found to increase the level of nitric oxide (NO), which plays a significant role in muscle repair and the reduction of inflammation [21]. Future research may require a more in-depth exploration of these mechanisms, taking into account individual variability factors, to formulate more personalized recovery strategies.

In this study, the results suggested that both acute and repeated IC could accelerate the recovery process, while repeated IC yielded better effects compared to acute application. This finding supported previous observations that 3 days of repeated IPC showed a better effect in MVIC recovery relative to acute administration [7]. This phenomenon could be related to the temporal effects of IC, which showed a biphasic pattern consisting of two distinct phases [28–30]. The initial phase occured immediately after IC and lasted for approximately 4 hours and was called the early phase [30]. Subsequently, the late phase began 12~24 hours post-IC and lasted for up to 48~72 hours [29, 31]. Given this temporal characteristic, the repeated use of IPC over five times could have cumulative effects. Interventions to facilitate player readiness could be especially crucial during seasons with concentrated competition and training time [7]. Whether the repeated application of IC could maintain optimal athletic performance and mitigate the risk of overtraining is worth investigating.

Several methodological considerations should be discussed. First, the results in our study were limited to healthy adults and might not be generalized to other populations, future research should explore the effects of IC in diverse populations, such as athletes with varying training backgrounds. This would provide a more comprehensive understanding of the applicability and effectiveness of IC across different demographic groups. Second, we only tested male participants, due to the reason that the studies of Teixeira [32] and Paradis-Deschenes [33] suggested that there might be gender differences in the effects of IC. Future research could compare the gender differences of IC. Third, participants’ macronutrient intake was not controlled. Dietary factors can significantly affect recovery and performance. Therefore, dietary differences may have influenced the results. Future studies should consider controlling participants’ diets to ensure more accurate and reliable outcomes. Another limitation of this study was the participants’ lack of prior experience with IC. This absence of experience might influence participants’ expectations and perceptions, potentially introducing placebo or nocebo effects. Finally, the IC protocol utilized in our study was standardized rather than individualized based on participants’ systolic blood pressure. It is noteworthy that without adjusting for individual systolic blood pressure, certain participants may have experienced over-occlusion. Future research should consider implementing a personalized protocol tailored to participants’ systolic blood pressure to address these limitations.

## CONCLUSIONS

In summary, this study provided valuable insights into the differential effects of acute and repeated IC interventions, highlighting their potential to promote recovery, reduce muscle damage, and modulate inflammatory and oxidative stress responses. The superiority of repeated IC, especially in the PEIC group, underscored its potential as an effective recovery strategy in the context of strength training.
